# Prevalence of depression in infertile men: a systematic review and meta-analysis

**DOI:** 10.1186/s12889-023-16865-4

**Published:** 2023-10-11

**Authors:** Zahra Kiani, Fahimeh Rashidi Fakari, Atena Hakimzadeh, Sepideh Hajian, Farzaneh Rashidi Fakari, Malihe Nasiri

**Affiliations:** 1 Midwifery and Reproductive Health Research Center, ShahidBeheshti University of Medical Sciences, Tehran, Iran; 2 Department of Midwifery, School of Nursing and Midwifery, Shahid Beheshti University of Medical Sciences, Tehran, Iran; 3 Student Research Committee, School of Nursing and Midwifery, Shahid Beheshti University of Medical Sciences, Tehran, Iran; 4 Department of Midwifery, School of Medicine, North Khorasan University of Medical Sciences, Bojnurd, Iran; 5 Department of Basic Sciences, School of Nursing and Midwifery, Shahid Beheshti University of Medical Sciences, Tehran, Iran

**Keywords:** Men, Infertility, Depression, Meta-analysis

## Abstract

**Background:**

Generally, infertile men hide their depression, which can threaten their health and lower their quality of life. Given the role of depression and its impact on people's health, this systematic review and meta-analysis aimed to investigate the prevalence of depression in infertile men.

**Methods:**

This research is a systematic review and meta-analysis based on preferred reporting items for systematic reviews and meta-analyses (PRISMA). Using the keywords of "Depression", "Emotional Depression", "Infertility", "Prevalence", and "Epidemiology", all English language articles were searched in international databases (PubMed, Cochran library, Web of sciences, Scopus, Embase, PsyINFO, and Google scholar) by two reviewers independently and without considering the time limit until September 2022. Title, abstract, full text and quality of each study were evaluated by two reviewers independently using the Newcastle–Ottawa Scale checklist. The results were analyzed using programming language and R software, and I^2^ test and Egger's Test were used to check heterogeneity and publication bias, respectively.

**Results:**

Twenty-two studies were included in the systematic part of this study; and 8 different measurement tools were used to identify depression. Then, based on the possibility of meta-analysis, 18 studies were included in 4 subgroups. Given the heterogeneity of the articles, random effect model was used. The overall prevalence of depression in infertile men was 18.30%. The lowest and highest overall prevalence of depression in men was reported to be 14.04% and 23.63% in the Zung Self-Rating Depression Scale (ZDS) and the Depression Anxiety Stress Scales (DASS) tools, respectively. The overall prevalence of depression among infertile men was reported to be 18.55% and 16.75% using the Beck Depression Inventory (BDI) and the Hospital Anxiety and Depression Scale (HADS) tools, respectively.

**Conclusion:**

Based on the findings of this study, the significant prevalence of depression in infertile men requires a specific attention and planning. The study revealed varying degrees of depression among infertile men, emphasizing the importance of assessing their mental health, specifically in terms of depression, during infertility treatments as a hidden variable. It is strongly recommended to develop training programs for health service providers to effectively utilize diagnostic tools in this particular field.

**Supplementary Information:**

The online version contains supplementary material available at 10.1186/s12889-023-16865-4.

## Introduction

Infertility refers to the failure of getting pregnant after 12 months of unprotected sex, which can be caused by either one of the couple [1]. Often it is impossible to determine the exact cause of infertility; such a disorder is defined as idiopathic infertility, which is identified with mental disorders such as stress, depression, sleep disorders, eating disorders, and addiction [2]. Although infertility is mostly the problem of less developed countries [3], approximately 8–12% of the world's population is infertile [4]. The total rate of infertility in Iran has been reported to be 13.2%, which is close to the global statistics [5]. Among the causes of infertility, male infertility (MI) has been observed in 50% of cases [6]. As one of the main problems of reproductive health, infertility is a serious issue for the World Health Organization, as insufficient attention to it in different countries has led to widespread psychological problems at the individual and social levels [7]. In other words, infertility has always caused various social, psychological, physical and financial stresses [8]. The incidence of emotional and mental disorders among infertile people is reported to be 25–60%, which is a significant value [9]; and among mental disorders of infertile people, anxiety and depression have been more important ones [10].

As a prevalent mental disorder, depression has involved approximately 121 million people worldwide. This mental disorder is usually identified with a number of signs and symptoms such as depressed mood, loss of interest or pleasure, feelings of guilt or worthlessness, sleep or appetite disturbances, low energy, and concentration difficulties [11]. Based on the estimations of the WHO in 2020, depression is considered as the second most common disease in the world [12]. The prevalence of depression in Iran has been reported to be 30.5% [13]. People who experience fertility problems suffer from anxiety and depression almost twice more than the general population [14]. In the study of Ogawa et al., the rate of depression in infertile men and women was 9.4% and 7.9%, respectively in Japan [15], however, Masoumi et al. reported 30.5% of depression among Iranian infertile couples [16]. In Gamel et al., severe level of depression was observed in 42% of infertile men and infertility had adverse consequences on their mental health in Egypt [14]. In study in Osmaniye, some degrees of depression were observed in 20% of infertile men, and 13.3% had clinical depression that required counseling. This study also indicated that the degree of depression in about one-third of infertile men was higher than that of healthy men, and the psychological burden caused by infertility could affect one's entire life [17].

All over the world, infertility is a stressful experience which affects couples psychologically, socially, personally and culturally [18]. Although the level of depression and anxiety in infertile women is said to be higher than infertile men [19], evidence has shown that psychiatric evaluation of both man and woman contributes to more efficient use of health services and increases the success of infertility treatment [20]. Therefore, psychiatric evaluation of men has been considered as an essential part of the treatment process. Given the fact that no study has hitherto meta-analyzed the prevalence of depression among infertile men, the present study aimed to investigate this issue based on published studies from different countries of the world.

## Methods

### Search Strategy

This study was conducted in international databases (PubMed, Cochran library, Web of sciences, Scopus, PsyINFO, Embase) and the Google scholar search engine by two researchers independently in English and without considering any time limit until September 2022. For an inclusive search in the above databases, keywords such as "Depression", "Emotional Depression ", "Infertility", "Prevalence", and "Epidemiology" were used. These keywords were combined with AND, OR operators and the specific search strategy of each database was used (search strategy,  Additional file 1).

#### Inclusion and exclusion criteria

First, male infertility was defined as lack of pregnancy after one year of intercourse without using any contraceptive method, according to the doctor's diagnosis or sperm analysis results. This was considered as one of the inclusion criteria for including each article in the study. Accordingly, studies with at least 30 samples were included in the initial review. Other inclusion criteria were as follows: cross-sectional studies, cross-sectional data from longitudinal studies, and studies which had used valid methods for evaluating depression (clinical interview or standard questionnaire). Thus, review articles, non-English articles, articles with non-human samples, case reports, mental illnesses, and articles whose full text was not available were excluded from the review.

#### Outcome measures

The main outcome in this study was depression in infertile men, which were assessed by standard tools (clinical interview or questionnaires) and reported the prevalence of depression in these men.

#### Data extraction

The data of the studies were extracted by two trained reviewers independently, and in case of disagreement, a third reviewer was asked to help in this regard. During the initial search, the articles were entered the EndNote software by two reviewers separately, and duplicate articles were removed.

The required data (e.g. name of authors, year of publication, place of research, sample size, type of infertility, prevalence of depression in infertile men, mean age, duration of infertility and type of tool) were extracted from the studies. The review steps are described in flowchart 1.

#### Quality evaluation

For quality assessment, we used the Newcastle–Ottawa Scale (NOS) checklist for assessing the quality if nonrandomized studies in meta-analyses, modified by Zhang et al. [19]. This checklist consists of 5 sections representativeness of the sample, sample size, non-respondents, ascertainment of anxiety and quality of descriptive statistics reporting. Accordingly, the articles that met the inclusion criteria were scored 0–5 based on the quality of the study and using NOS. Then, based on the total scores of < 3 and ≥ 3, the articles were classified respectively as high-risk and low-risk studies in terms of their quality (Table 1). In this study, quality assessment was done by two reviewers independently, and in case of disagreement, the opinions of a third reviewer were also used (Additional file 2). This systematic study was reported based on preferred reporting items for systematic reviews and meta-analyses (PRISMA) [18].

**Table 1:** Characteristics of the studies selected for the meta-analysis

ID	Authors	Year publication	countries	Income levels	Sample size	Type of infertility	Prevalence of depression	Age (Y) (mean ± SD)	Mean years of infertility (mean ± SD)	Type of Tools	Quality assessment
1	Alosaimi et al. [20]	2015	Saudi Arabia	High	200	Primary and secondary	26.20%	NA	5.4 ± 4.3	MINI	3/5
2	Öztekin et al. [21]	2020	Turkey	Low and middle	130	Primary and secondary	21.26%	29.95 ± 4.73	2.1 8 ± 16.5	BDI	3/5
3	Öztekin et al. [22]	2015	Turkey	Low and middle	48	Primary and secondary	20.13%	35.6 ± 3.7	1.8 ± 3.5	BDI	3/5
4	Gamel et al. [7]	2019	Egypt	Low and middle	50	Primary and secondary	40.12%	39 ± 13.84	-	DASS	3/5
5	Peterson et al. [23]	2006	USA	High	506	Primary and secondary	14%	33.9 ± 5.4	3.4 ± 0.0	BDI	4/5
6	Noorbala et al. [24]	2008	Iran	Low and middle	319	Primary and secondary	23.8%	36.5 ± 3.6	4.6 ± 1.5	BDI	4/5
7	Peterson et al. [25]	2003	USA	High	506	Primary and secondary	9.0%	33.8 ± 0.0	NA	BDI	4/5
8	Maroufizadeh et al. [26]	2015	Iran	Low and middle	122	Primary and secondary	20.0%	33.9 ± 5.1	6.2 ± 4.1	HADS	3/5
9	Maroufizadeh et al. [27]	2018	Iran	Low and middle	479	Primary and secondary	31.7%	34.53 ± 5.69	5.62 ± 4.03	HADS	4/5
10	Beutel et al. [28]	1999	Germany	High	281	Primary and secondary	12%	35.9 ± 0.0	1.9 ± 4.3	D-S	4/5
11	Faramarzi et al. [29]	2013	Iran	Low and middle	168	Primary and secondary	30%	30.4 ± 5.1	4.6 ± 3.5	BDI	3/5
12	Liu et al. [30]	2021	China	Low and middle	247	Primary and secondary	13.77%	31.72 ± 4.86	3.1 ± 1.9	ZDS	4/5
13	Li et al. [31]	2013	China	Low and middle	844	Primary and secondary	13.5%	33.8 ± 5.1	2.6 ± 3.1	CES-D	4/5
14	Kazemi et al. [32]	2021	Iran	Low and middle	212	Primary and secondary	20.1%	36.7 ± 5.0	6.0 ± 4.3	DASS	4/5
15	Chiaffarino et al. [33]	2011	Italy	High	872	Primary and secondary	6.9%	38.55 ± 7.59	NA	ZDS	4/5
16	Drosdzol and Skrzypulec [34]	2009	Poland	High	188	Primary and secondary	15.60%	32.1 ± 5.60	NA	BDI	4/5
17	Fernande et al. [35]	2023	Portugal	High	107	Primary and secondary	3.2%	35.5 ± 5.50	9.19 ± 5.01	HADS	3/5
18	Kissi et al. [36]	2013	Tunisia	Low and middle	100	Primary and secondary	23.5%	38.74 ± 5.87	5.19 ± 4.62	HADS	3/5
19	Babore et al. [37]	2017	Italy	High	170	Primary and secondary	24.1%	39.37 ± 5.75	3.29 ± 5.71	ZDS	4/5
20	Yang et al. [38]	2017	China	Low and middle	711	Primary and secondary	20.8%	37.8 7 ± 6.29	2.15 ± 48	MHI-5	4/5
21	Hegyi et al. [39]	2019	Hungary	High	113	Primary	23.1%	33.3 ± 3.5	2.5 ± 6.5	BDI	3/5
22	Musa et al. [40]	2014	Malaysia	Low and middle	123	Primary and secondary	16.80%	NA	NA	DASS	4/5

*Abbreviations*: *NA* Not reported, *MINI* Mini International Neuropsychiatric Interview, *HADS* Hospital Anxiety and Depression Scale, *ZDS* Zung Self-Rating Depression Scale, *BDI* Beck depression inventory, *DASS* Depression Anxiety Stress Scales, *CES-D* Center of Epidemiologic Studies Short Depression Scale, *MHI-5* Mental Health Inventory–5, D-S (Depression Scale): The von Zerssen Depression Scale

#### Statistical analysis

In this study, I^2^ index was used to check the heterogeneity of the studies. Egger's test was also used to check publication bias. The overall prevalence of depression in infertile men was performed. Subgroup analysis was performed based on the type of tool. The data were analyzed using programming language and software. The significance level for statistical tests was considered to be 0.05.

### Ethics approval and consent to participate

Ethics approval was obtained from the Ethics Committee, Faculty of Pharmacy and Nursing.

Midwifery, Shahid Beheshti University (Ethical code: IR.SBMU.RETECH.REC.1401.288). All methods were carried out in accordance with relevant guidelines and regulations.

## Results

**Fig. 1:**
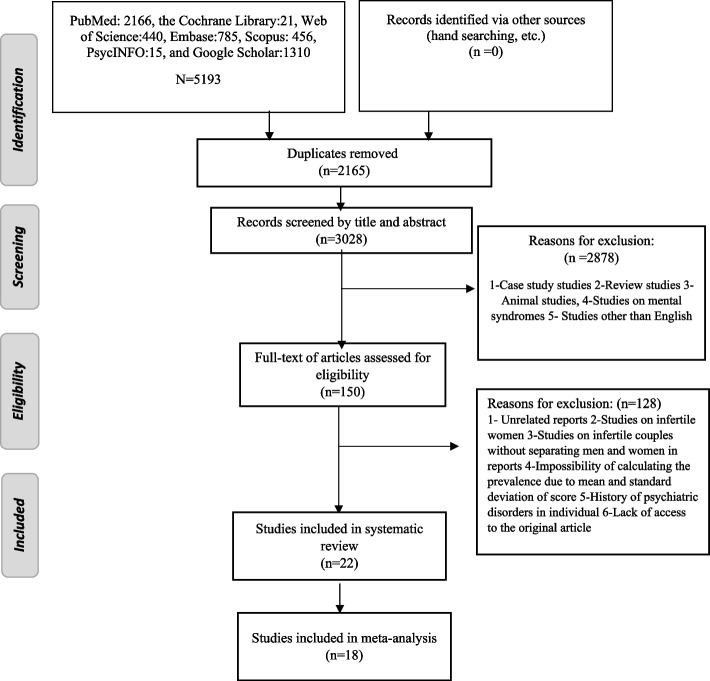
Flowchart for selection of studies

Initially 5,193 articles were found. After removing the duplicate articles, the title and abstract of the remaining articles were reviewed, and after discarding the articles unrelated to the purpose of the research and considering the inclusion and exclusion criteria, the original text of 150 articles was further reviewed. Finally, 22 articles entered the final study and were analyzed (Fig. 1).

The total sample size of the studies was 6,496 infertile men, and the smallest and the largest sample sizes were 48 and 872 subjects, respectively. The lowest and highest prevalence rates were 3.2% and 40.12% which were reported in Portugal and Egypt, respectively. The studies were conducted in 9 high-income and 13 low- and middle-income countries. One study used Mini International Neuropsychiatric Interview (MINI) [20], 3 studies the Depression Anxiety Stress Scales (DASS) [14, 32, 40], one study the Center of Epidemiologic Studies Short Depression Scale (CES-D) [31], one study the Mental Health Inventory–5 (MHI-5) [38], one study Depression Scale (D-S) [28], 3 studies the Zung Self-Rating Depression Scale (ZDS) [33, 37, 41], 4 studies the Hospital Anxiety and Depression Scale (HADS) [26, 27, 36, 42] and 8 studies the Beck Depression Inventory (BDI) [21–25, 29, 34, 39] to identify depression (Table 1).

### Types of tools

#### MINI

The MINI is a short and structured diagnostic interview that was originally developed in the 1990s by psychiatrists and clinicians in the United States and Europe to assess DSM-III-R and ICD-10 psychiatric disorders [43]. It is widely recognized and utilized as part of the clinical evaluation process for depression and anxiety in primary care [44]. The MINI is considered the most effective structured psychometric diagnostic interview tool globally, and it is employed by mental health professionals and healthcare organizations in more than 100 countries. Numerous studies have confirmed its validity and reliability [45–48].

#### HADS

HADS is a 14-item scale with each seven items for anxiety and depression subscales. Each item is scored on a scale ranging from zero to three [49]. The total scores for each subscale range from 0 to 21 (0 to 7 indicating normal, 8 to 10 mild, 11 to 14 moderate, and 15 to 21 severe) [50]. The HADS offers several advantages, including its brevity, ease of scoring, and relatively high sensitivity. Its reliability and validity have been confirmed through studies conducted in various countries worldwide [51–53].

#### ZDS

The ZDS is a short self-administered survey used to measure the level of depression in a patient [54]. It was developed by Zung to assess depression severity [55]. Zung has reported a split-half reliability coefficient of 0.73, which has been confirmed through various studies examining its validity and reliability [54, 56, 57] The questionnaire consists of 20 items rated on a Likert scale from 1 to 4, and the total score ranges from 20 to 80 (20 to 44 indicating normal, 45 to 59 mild, 60 to 69 moderate, and 70 and above severe) [58].

#### BDI

The BDI consists of 21 items designed to evaluate various symptoms of depression [59]. There are different versions of this tool [60]. The total score on the BDI ranges from 0 to 63, with higher scores indicating greater levels of depression (0 to 9 indicating no symptoms, 10 to 18 mild, 19 to 29 moderate, and 30 to 63 severe) [61]. The BDI has demonstrated high construct validity in relation to the medical symptoms it measures. Studies have reported coefficient alpha values ranging from 0. 8 to above 0. 90 [62–65].

#### DASS

The 21-item DASS was developed by Lavibond and Lavibond in 1995 to assess stress-anxiety-depression [66]. The questionnaire comprises three components, with each subscales containing 7 items. The final score for each subscale is calculated by summing the scores of the corresponding items. Each question is scored from zero to 3. Since the DASS-21 is a shortened version of the original scale (which had 42 items), the final score for each subscale should be doubled [67]. The validity and reliability of the DASS-21 have been established, and its usefulness has been supported in both public and clinical settings [68–70].

#### CES-D

The CES-D was developed by Rudolph to assess depressive symptoms in the general population. This 20-item questionnaire is combined of different questionnaires [71]. Each item is scored on a scale from 0 to 3, and the maximum possible score is 60. Higher scores, particularly above 16, indicate a need for further clinical evaluation to diagnose mood disorders [72, 73]. The CES-D demonstrates adequate screening sensitivity and specificity when used in the general population or primary care settings. However, it should not be solely relied upon as a diagnostic measure for depression. Depending on the test objectives, a cut-off score of 20 may be more appropriate than the value of 16, which is suggested [72].

#### MHI-5

The MHI-5 is a concise, valid, and reliable universal tool for evaluating mental health. It was established as part of the National Health Insurance Study. The MHI-5 consists of five items, and each item is rated on a Likert scale ranging from 1 to 6. The minimum and maximum scores are 5 and 30 for each person. This is then transformed into a variable ranging from 0–100 using a standard linear transformation [74]. The MHI-5 has been supported by evidence regarding its validity and reliability [75–78].

#### D-S

The D-S is used to measure fearful and irritable depression. It consists of 16 items that are rated on a 4-point scale, ranging from ‘not true’ to ‘completely true’ [28]. This tool evaluates the symptoms of depression and anxiety and its scores range from 0 and 48 [79].

#### Evaluation of heterogeneity and meta-analysis

**Fig. 2:**
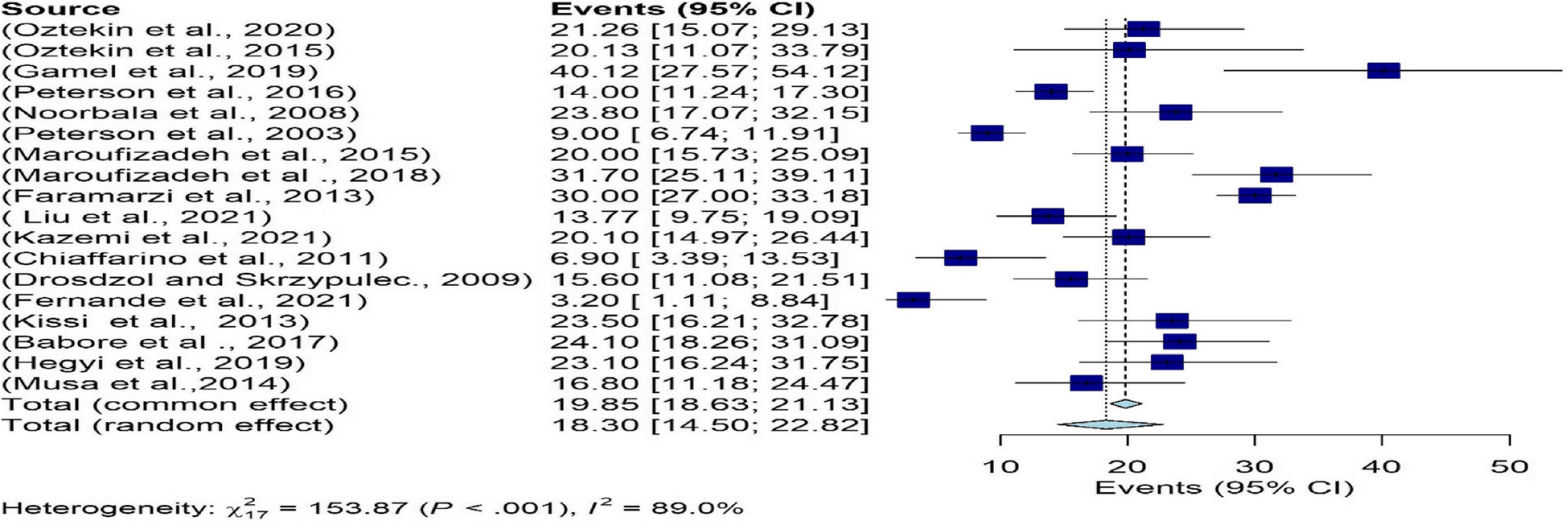
The overall prevalence of depression in infertile men

**Fig. 3:**
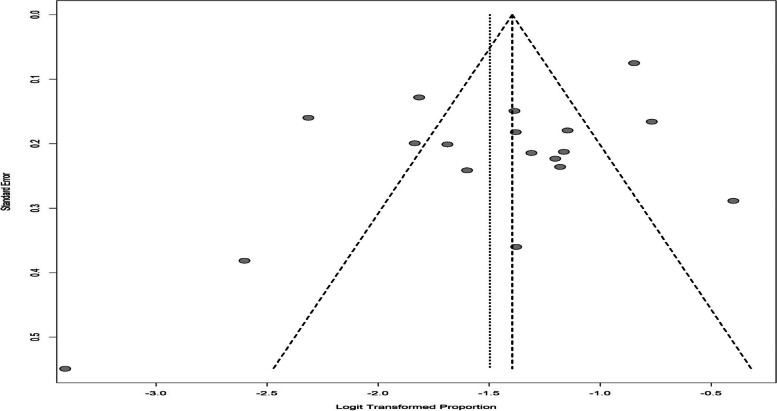
Funnel chart of overall prevalence of depression in infertile men

Given the fact that different tools were used to investigate depression in infertile men, from the 22 articles of the systematic review section, 18 studies were used for meta-analysis in 4 subgroups of DASS, ZDS, HADS, and BDI tools. The I^2^ index for investigating heterogeneity was greater than 50%, and the results of the random effects method were used for reporting. The overall prevalence of depression in infertile men was found to be 18.30% (95%CI: 14.50–22.82) (Fig. 2). The results of the funnel chart for assessing the overall prevalence of depression and the results of Egger's test (t = -2.00, df = 16, *p*-value = 0.062) which indicate the absence of diffusion bias are also shown in Fig. 3.

**Fig. 4:**
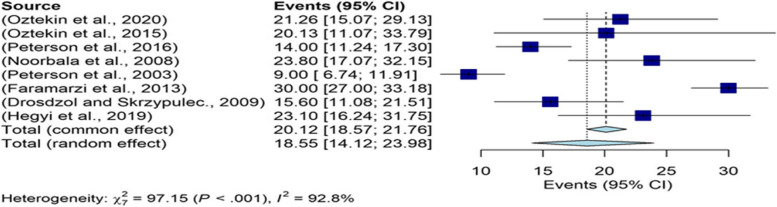
The overall prevalence of depression in infertile men in subgroup of BDI tool

Based on the analysis in the subgroup of BDI tool, the overall prevalence of depression in men was 18.55% (95%CI: 14.12–23.98) (Fig. 4).

**Fig. 5:**
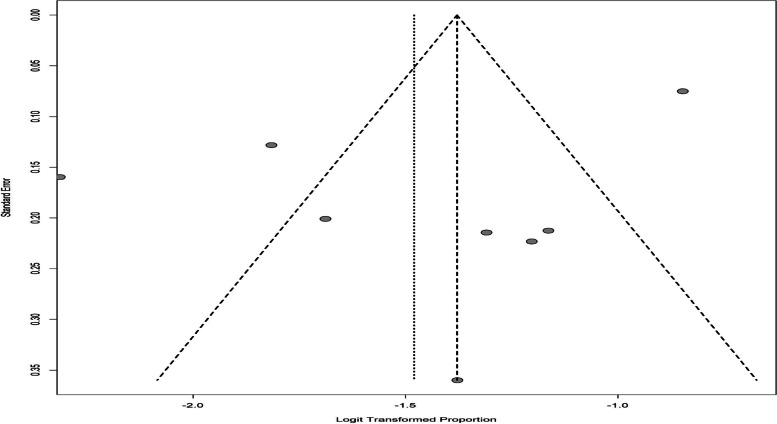
Funnel chart based on the BDI subgroup

The results of the funnel chart and the results of Egger's test (t = -1.34, df = 6, *p*-value = 0.2300) which indicate the absence of diffusion bias are also shown in Fig. 5.

**Fig. 6:**
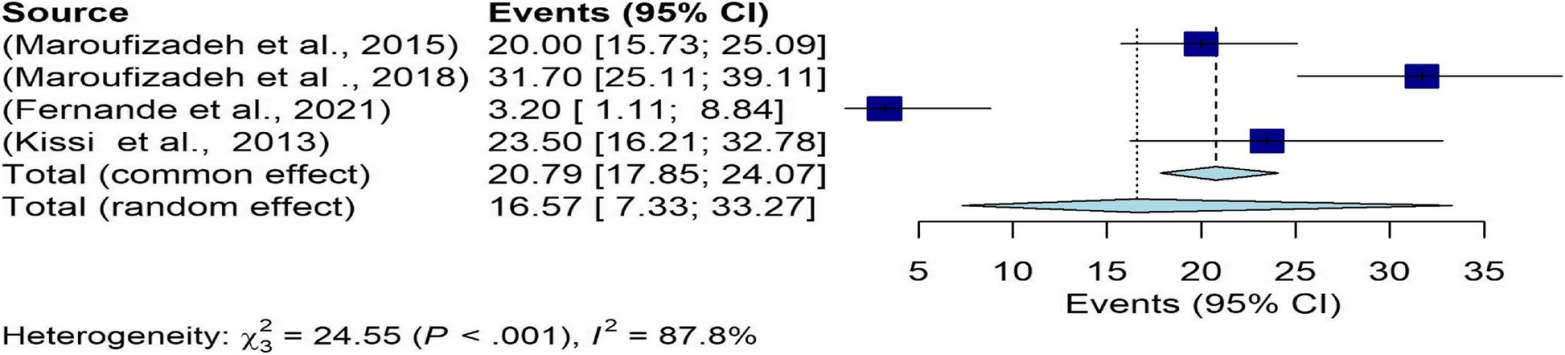
The overall prevalence of depression in infertile men in the HADS subgroup

**Fig. 7:**
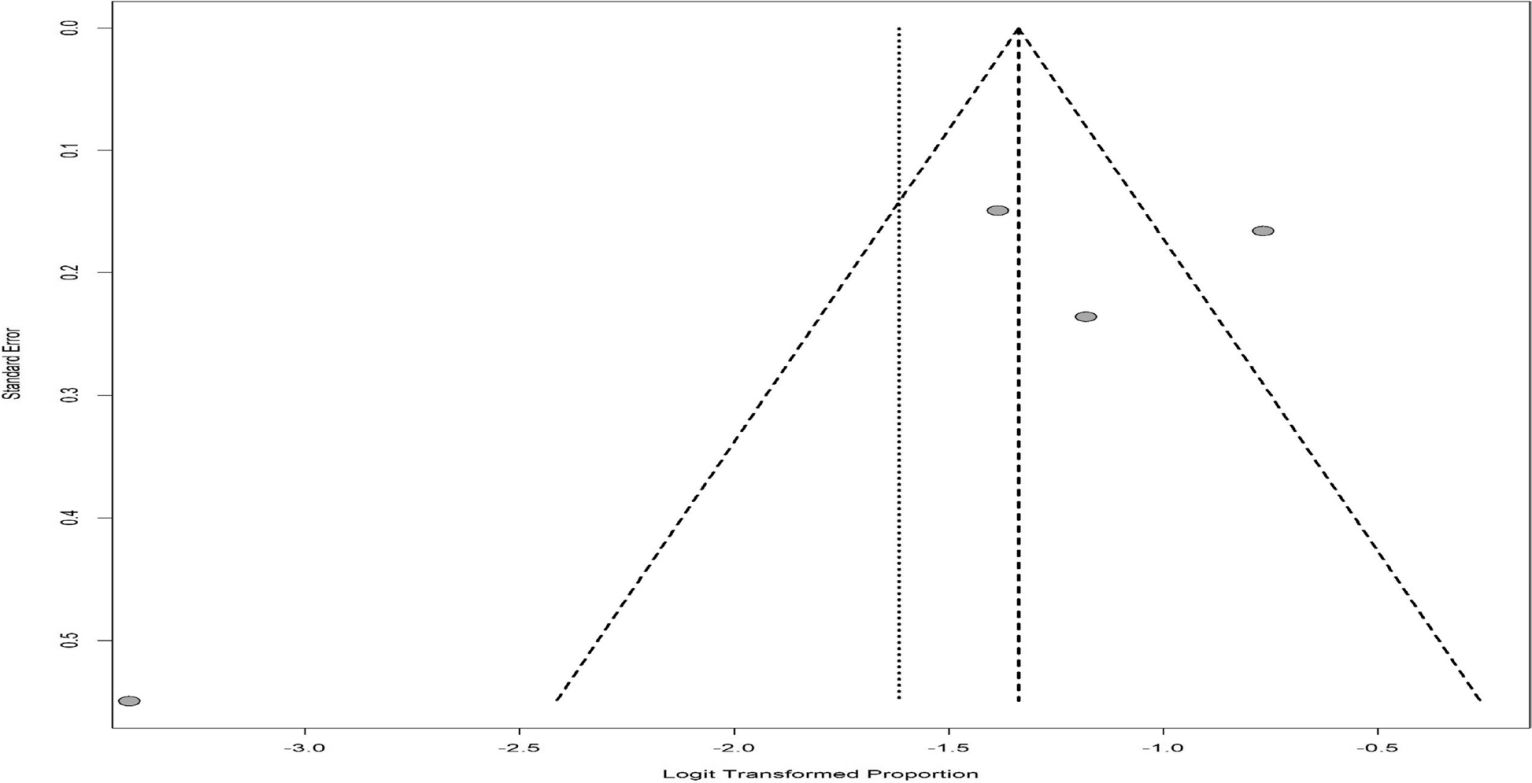
Funnel chart based on the HADS subgroup

Based on the HADS subgroup results, the overall prevalence of depressive in infertile men was 16.57% (95% CI: 7.3–33.27) (Fig. 6). The results of the funnel chart are also shown in Fig. 7.

**Fig. 8:**
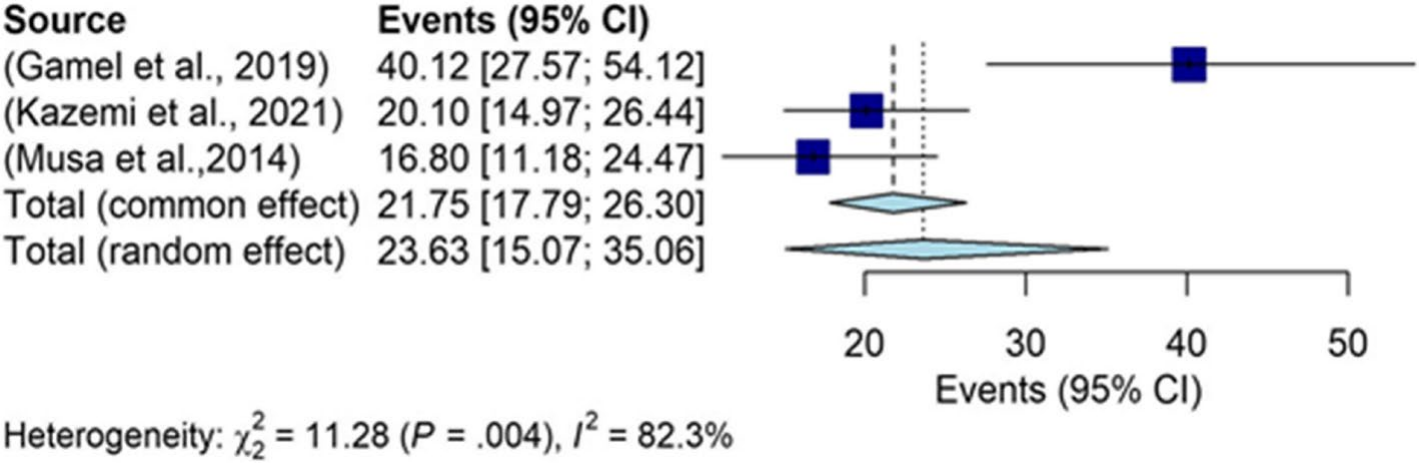
The overall prevalence of depression in infertile men in the DASS subgroup

**Fig. 9:**
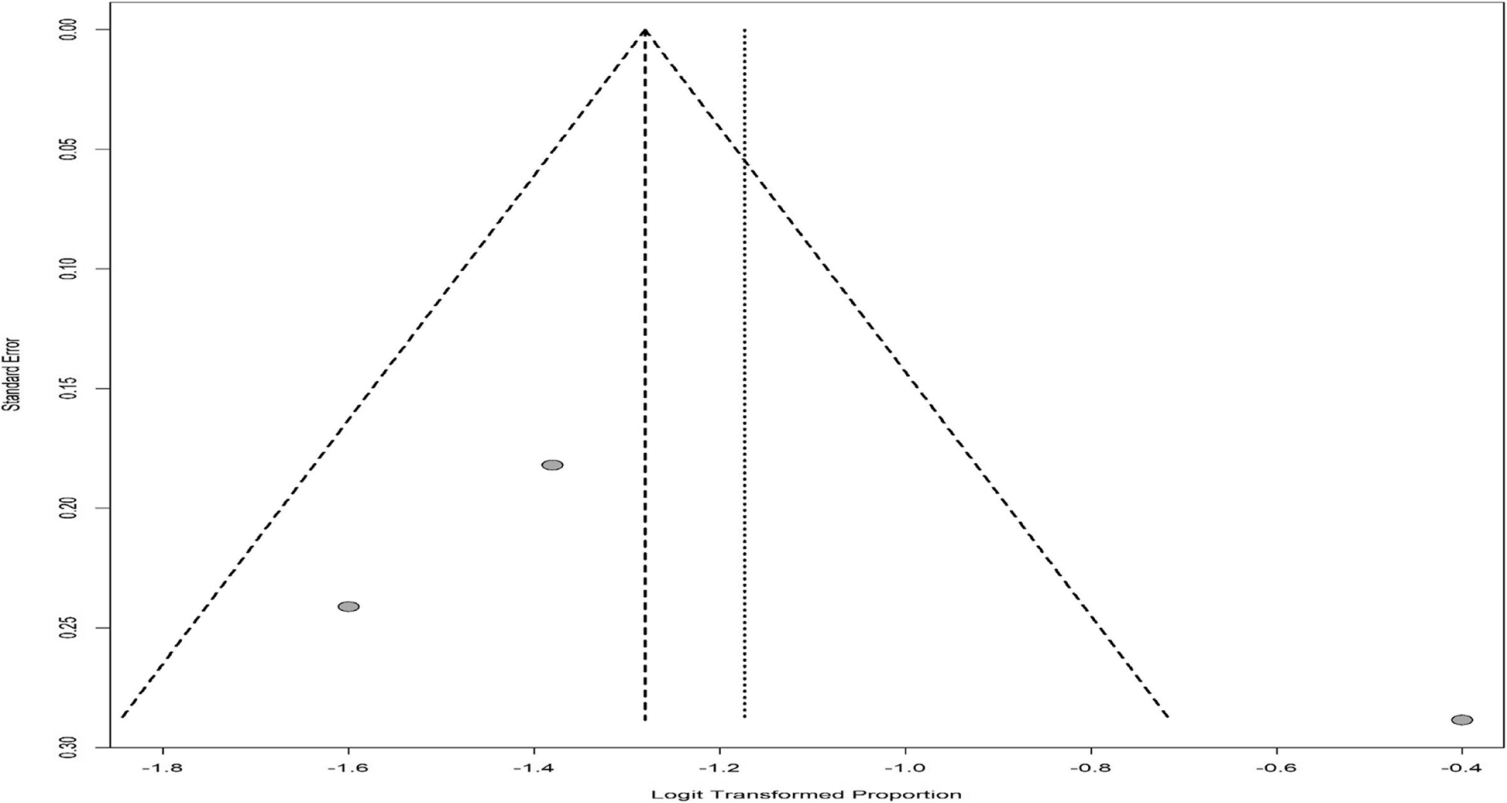
The funnel chart based on the DASS subgroup

The results of the DASS subgroup were indicative of the overall prevalence of 23.63% (95% CI: 15.07–35.06) in the depression of infertile men (Fig. 8), which was the greatest prevalence in our study. The funnel chart is shown in Fig. 9.

**Fig. 10:**
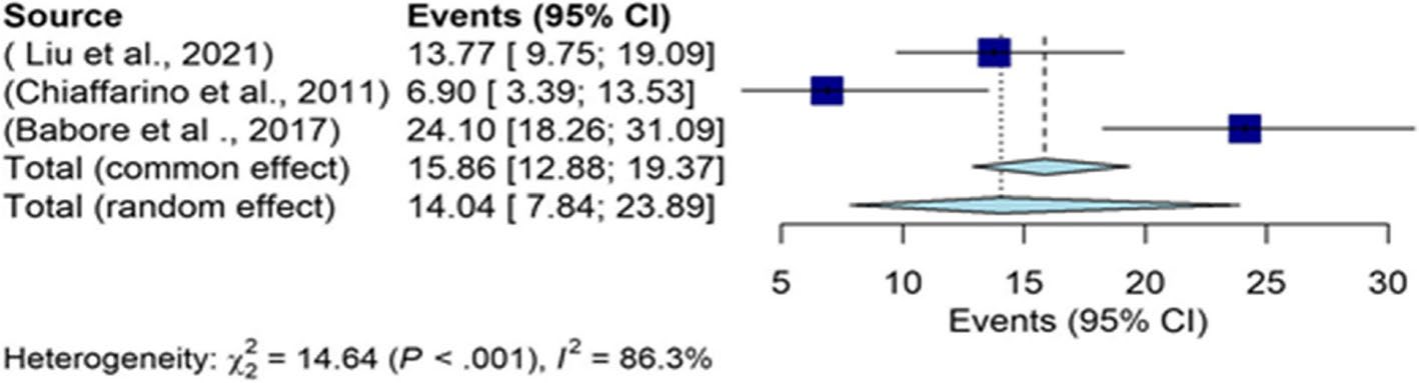
The overall prevalence of depression in infertile men in the ZDS subgroup

**Fig. 11:**
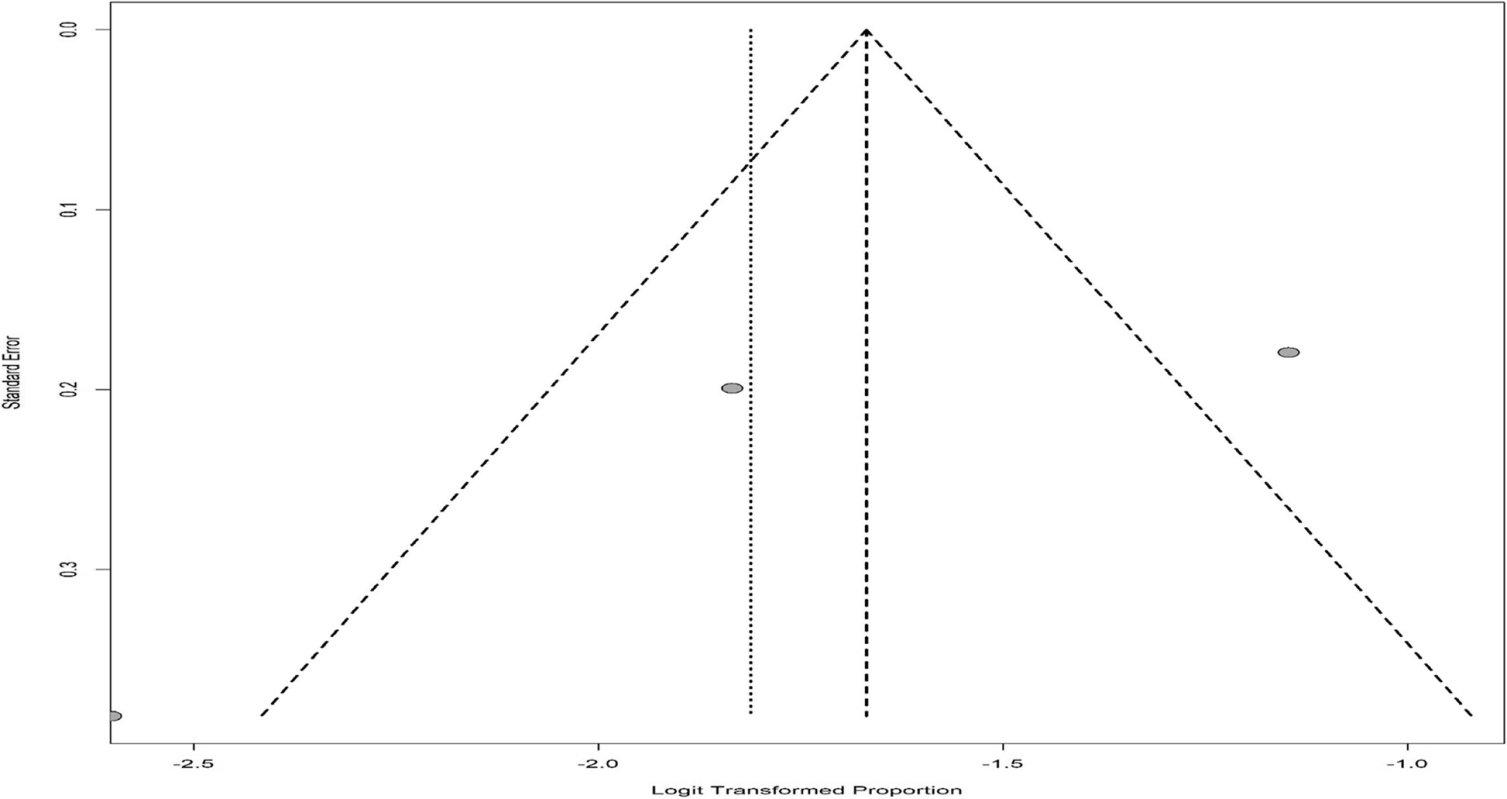
Funnel chart based on the ZDS subgroup

The results of the ZDS subgroup were indicative of the overall prevalence of 14.04% (95% CI: 7.84–23.89) in the depression of infertile men (Fig. 10), which was the lowest prevalence of depression in men based on this tool. The funnel chart is shown in Fig. 11.

## Discussion

The present study aimed to investigate the prevalence of depression in infertile men. Based on the results of this meta-analysis, the lowest and highest prevalence of depression were 14.04% and 23.63%, which were based on the ZDS and DASS tools.

The overall prevalence of depression in infertile women has been reported to be 21–52% [80] which, considering the family and social pressures tolerated by infertile women [81], the depression can be expected in this population of women. The mental health of infertile men is an important aspect that should not be overlooked. In a meta-analysis, the prevalence of male depression in the general population was reported to be 2.3% [82]. However, in the present study, the prevalence of depression among infertile men was found to be higher, ranging from 14 to 23%.Furthermore, a comparative study revealed that depression was reported in infertile men across different age groups [83]. Another study found that infertile men, when compared to men in the control group, had lower scores in emotional, mental, and social aspects of quality of life [84], which suggests a potential vulnerability to psychological disorders. These findings highlight the undeniable impact of infertility on the mental health of men. However, less attention has been paid to the psychological aspects of infertile men and their mental health is usually overlooked [85].

This inattention to the emotional reactions of infertile men can be partly related to men's insufficient information and their avoidance of talking about the issue of infertility with others. Moreover, men usually do not want to seek help from mental health professionals [86]. It was reported in a study that infertile men prefer to receive even psychological support from infertility therapists and specialists [87]. Another part of this lack of attention to the psychological dimensions of infertile men can be related to the healthcare system as the psychological dimensions of infertile men are less considered in the treatment processes. In a study, 63% of infertile men stated that specialists usually interact more with their wives and demanded to be seen equally [88].

Therefore, not only infertile women are prone to depression, but infertile men are also affected by psychological pressure, as they are the main and sometimes the only source of family income. Furthermore, infertility treatments are expensive and impose a double psychological burden on infertile couples [89]. It was reported in a study that the financial burden of infertility treatments threatens occupational and financial status of men as the main providers of treatment costs [90]. All of these make infertile men also prone to more mental distress.

In addition to economic problems, social and cultural issues also seem to affect men's psychological aspects. In some countries, where masculinity is equivalent to fertility, male infertility is considered to be a stigma. In such societies, men are exposed to more psychological distresses, which make them prone to depression [38, 91]. In the study of Ahmadi et al. (2011), the prevalence of depression in Iranian infertile men was estimated to be about 43% [90], meaning that almost half of infertile men might be depressed. By contrast, in the study of Fernandes et al. (2021), the lowest prevalence of depression (3%) was reported among those subjects of this meta-analysis who suffered the longest period of infertility in Portugal [35]. While the prevalence of depression is expected to be increased with the duration of infertility [92], social and cultural issues may not also be unaffected. In fact, different view toward male fertility in the context of some countries creates a greater urgency for fertility even at younger ages. Therefore, the contribution of social and cultural issues in fertility behaviors, which definitely have an undeniable effect on mental health, should not be overlooked.

By the way, as a substantial psychological disorder in infertile men [2], depression affects not only mental health, but also treatment outcomes [38, 91]. Fertility outcomes will also be improved by informing men about psychological consequences [93], and using appropriate interventions [94, 95]. Another important point is that knowing the prevalence rates of depression in infertile men makes specialists more alert to the assessment of mental distress, even in the initial visits or the screening stage. This, in turn, results in faster diagnosis makes treatment outcomes be fulfilled in a shorter time and increases the probability of success.

In all studies reviewed in the present meta-analysis, various questionnaires were used to diagnose depression in infertile men. Although the differences between the questionnaires were not significant, the BDI could be recommended as an initial diagnostic tool for depression in infertile men. This is because the BDI was used in a larger number of studies, and its focus is specifically on the variable of depression. It should be noted that a relationship between cytokine biomarkers and depression in infertile men has been reported, which also affects fertility outcomes [96]. Therefore, incorporating biomarkers into the diagnostic process may help strengthen the diagnosis of psychological disorders. However, compared to biomarkers, the use of questionnaires is more affordable, readily available and provides a faster diagnosis process.

One of the limitations of the present study was the inclusion of studies with different tools in the measurement of depression, as a result of which it was almost impossible to combine all studies at once, though all scales were of the same type (subjective). Another limitation of the study was that non-English studies were not included, and the full text of some articles was not available. One of the strengths of the study was addressing the prevalence of psychological aspects of infertility, especially in men, which have been less addressed before.

## Conclusion

Based on the results of the present meta-analysis, the prevalence of depression in men was 14–23%, which should not be overlooked. Accordingly, infertility specialists need to pay more attention on the psychological aspects of infertile men as well. In this regard, focusing on early screenings for men's depression during therapeutic interventions is needed to achieve better fertility outcomes. Therefore, it is recommended to develop educational packages or retraining programs for healthcare providers to improve their recognition and utilization of tools for diagnosing depression in men. These programs should be designed to consider the cultural and social context in which the healthcare providers operate.

### Supplementary Information

**Additional file 1:** **Appendix 1.** Search Strategy.

**Additional file 2:** **Appendix 2.** Quality assessment.

## Data Availability

All data related to this review is included in the result section of the manuscript. If any further data is needed it can be accessible via the corresponding author on request.

## Abbreviations

MI: Male Infertility
WHO: World Health Organization
NOS: The Newcastle–Ottawa Scale
PRISMA: Preferred reporting items for systematic reviews and meta-analyses
NA: Not reported
MINI: Mini International Neuropsychiatric Interview
HADS: Hospital Anxiety and Depression Scale
ZDS: Zung Self-Rating Depression Scale
BDI: Beck depression inventory
DASS: Depression Anxiety Stress Scales
CES-D: Center of Epidemiologic Studies Short Depression Scale
MHI-5: Mental Health Inventory–5
D-S: Depression Scale
