# Supplementary material for: Prevalence of depression in infertile men: a systematic review and meta-analysis
Source: BMC Public Health. 2023 Oct 11;23:1972. doi: 10.1186/s12889-023-16865-4 (PMC10568846; doi:10.1186/s12889-023-16865-4)
Supplement: Supplementary file 1 — Additional file 1: Appendix 1. Search Strategy. [file 12889_2023_16865_MOESM1_ESM.docx]

**Appendix 1: Search Strategy**

| **Pubmed** |
| --- |
| (((((((((Infertility[Title/Abstract]) OR (Sterility, Reproductive[Title/Abstract])) OR (Sterility[Title/Abstract])) OR (Reproductive Sterility[Title/Abstract])) OR (Subfertility[Title/Abstract])) OR (Sub-Fertility[Title/Abstract])) OR (((((((((Infertility, Male[Title/Abstract]) OR (Male Infertility[Title/Abstract])) OR (Sterility, Male[Title/Abstract])) OR (Male Sterility[Title/Abstract])) OR (Subfertility, Male[Title/Abstract])) OR (Male Subfertility[Title/Abstract])) OR (Sub-Fertility, Male[Title/Abstract])) OR (Male Sub-Fertility[Title/Abstract])) OR (Sub Fertility, Male[Title/Abstract]))) AND ((((((Depression[Title/Abstract]) OR (Depressive Symptoms[Title/Abstract])) OR (Depressive Symptom[Title/Abstract])) OR (Symptom, Depressive[Title/Abstract])) OR (Emotional Depression[Title/Abstract])) OR (Depression, Emotional[Title/Abstract]))) OR ((((((((Prevalence[Title/Abstract]) OR (Prevalences[Title/Abstract])) OR (Period Prevalence[Title/Abstract])) OR (Period Prevalences[Title/Abstract])) OR (Prevalence, Period[Title/Abstract])) OR (Point Prevalence[Title/Abstract])) OR (Point Prevalences[Title/Abstract])) OR (Prevalence, Point[Title/Abstract]))) OR (((((Epidemiology[Title/Abstract]) OR (Social Epidemiology[Title/Abstract])) OR (Epidemiologies, Social[Title/Abstract])) OR (Epidemiology, Social[Title/Abstract])) OR (Social Epidemiologies[Title/Abstract]))  **N=2166** |
| **Scopus** |
| (TITLE-ABS-KEY("Prevalence “) OR (TITLE-ABS-KEY (Epidemiology) AND TITLE-ABS-KEY (Infertility)) OR TITLE-ABS-KEY(Sterility) OR TITLE-ABS-KEY("Reproductive Sterility “) OR TITLE-ABS-KEY (Reproductive Sterility) OR (TITLE-ABS-KEY (Male Infertility) AND TITLE-ABS-KEY(Depression)) OR TITLE-ABS-KEY(Depressive Symptoms)) OR TITLE-ABS-KEY(Emotional Depression)  **N= 456** |
| **ISI** |
| (((TS=(Infertility OR Sterility, Reproductive OR Sterility OR Reproductive Sterility OR Subfertility OR Sub-Fertility )) OR TS=(Infertility, Male OR Male Infertility OR Male Sterility OR Sterility, Male OR Subfertility, Male OR Sub-Fertility, Male OR Male Subfertility )) AND TS=(Depression OR Depressive Symptoms OR Depressive Symptom OR Symptom, Depressive OR Emotional Depression OR Depression, Emotional )) AND TS=(Prevalence OR Epidemiology)  **N=440** |
