# Supplementary material for: Prevalence of depression in infertile men: a systematic review and meta-analysis
Source: BMC Public Health. 2023 Oct 11;23:1972. doi: 10.1186/s12889-023-16865-4 (PMC10568846; doi:10.1186/s12889-023-16865-4)
Supplement: Supplementary file 2 — Additional file 2: Appendix 2. Quality assessment. [file 12889_2023_16865_MOESM2_ESM.docx]

**Appendix 2**: Quality Assessment

Modified Newcastle-Ottawa scoring guide.

**(1) Representativeness of the sample:**

1 point: Population contained a mixture of specialties at multiple sites.

0 points: Population contained a single specialty at a single site.

**(2) Sample size:**

1 point: Sample size was 200 and greater than 200 participants.

0 points: Sample size was less than 200 participants or a convenience sample.

**(3) Non-respondents:**

1 point: Comparability between respondent and non-respondent characteristics was established, and the response rate was satisfactory.

0 points: The response rate was unsatisfactory, the comparability between respondents and non-respondents was unsatisfactory, or there was no description of the response rate or the characteristics of the responders and the non-responders.

**(4) Ascertainment of depression:**

1 point: Validated measurement tool using a validated cutoff score or clinical interview.

0 points: Non-validated measurement tool, or validated measurement tool with non-valid cutoff score, or 2-item PRIME-MD (scored as such due to its low specificity).

**(5) Quality of descriptive statistics reporting:**

1 point: Reported descriptive statistics to describe the population (*e.g.*, age, sex) with proper measures of depression (*e.g.*, standard deviation, standard error, range, percentage).

0 points: Descriptive statistics were not reported, were incomplete, or did not include proper measures of depression.

**Legend:** This scale, the scoring of which ranges from 0 to 5, assesses quality in several domains: sample representativeness and size, comparability between respondents and non-respondents, ascertainment of depressive symptoms, and statistical quality. Studies were judged to be of low risk of bias (≥3 points) or high risk of bias (<3 points).

**Total = /5**

|  | **Results of Newcastle-Ottawa Risk of Bias Assessment** | | | | | | |
| --- | --- | --- | --- | --- | --- | --- | --- |
|  | **Study ID** | **Representativeness** | **Size** | **Comparability** | **Outcome** | **Statistics** | **Total** |
| 1 | Authors | **0** | **1** | **0** | **1** | **1** | **3** |
| 2 | Alosaimi et al. ([21](#_ENREF_21)) | **1** | **0** | **0** | **1** | **1** | **3** |
| 3 | [Öztekin](https://www.ncbi.nlm.nih.gov/pubmed/?term=%26%23x000d6%3Bztekin%20%26%23x000dc%3B%5BAuthor%5D&cauthor=true&cauthor_uid=32252512) et al ([22](#_ENREF_22)) | **1** | **0** | **0** | **1** | **1** | **3** |
| 4 | [Öztekin](https://www.ncbi.nlm.nih.gov/pubmed/?term=%26%23x000d6%3Bztekin%20%26%23x000dc%3B%5BAuthor%5D&cauthor=true&cauthor_uid=32252512) et al ([23](#_ENREF_23)) | **0** | **1** | **0** | **1** | **1** | **3** |
| 5 | Gamel et al ([7](#_ENREF_7)) | **1** | **1** | **0** | **1** | **1** | **4** |
| 6 | Peterson et al ([24](#_ENREF_24)) | **1** | **1** | **0** | **1** | **1** | **4** |
| 7 | Noorbala et al ([25](#_ENREF_25)) | **1** | **1** | **0** | **1** | **1** | **4** |
| 8 | Peterson et al([26](#_ENREF_26)) | **0** | **1** | **0** | **1** | **1** | **3** |
| 9 | Maroufizadeh et al([27](#_ENREF_27)) | **1** | **1** | **0** | **1** | **1** | **4** |
| 10 | Maroufizadeh et al ([28](#_ENREF_28)) | **1** | **1** | **0** | **1** | **1** | **4** |
| 11 | Beutel et al ([29](#_ENREF_29)) | **0** | **0** | **1** | **1** | **1** | **3** |
| 12 | Faramarzi et al ([30](#_ENREF_30)) | **1** | **1** | **0** | **1** | **1** | **4** |
| 13 | [Liu](https://www.ncbi.nlm.nih.gov/pubmed/?term=Liu%20YF%5BAuthor%5D&cauthor=true&cauthor_uid=33658786) et al ([31](#_ENREF_31)) | **0** | **1** | **1** | **1** | **1** | **4** |
| 14 | Li et al ([32](#_ENREF_32)) | **1** | **1** | **0** | **1** | **1** | **4** |
| 15 | Kazemi et al ([33](#_ENREF_33)) | **0** | **1** | **1** | **1** | **1** | **4** |
| 16 | Chiaffarino et al ([34](#_ENREF_34)) | **0** | **1** | **1** | **1** | **1** | **4** |
| 17 | Drosdzol and Skrzypulec ([35](#_ENREF_35)) | **1** | **0** | **0** | **1** | **1** | **3** |
| 18 | Fernande et al ([36](#_ENREF_36)) | **0** | **0** | **1** | **1** | **1** | **3** |
| 19 | Kissi et al ([37](#_ENREF_37)) | **1** | **1** | **0** | **1** | **1** | **4** |
| 20 | Babore et al ([38](#_ENREF_38)) | **0** | **1** | **1** | **1** | **1** | **4** |
| 21 | Yang et al. ([39](#_ENREF_39)) | **1** | **0** | **0** | **1** | **1** | **3** |
| 22 | Hegyi et al. ([40](#_ENREF_40)) | **0** | **1** | **1** | **1** | **1** | **4** |
